# Hyperbaric Oxygen Therapy for PTSD: Threshold Effect for Sustained Symptom Improvement in a Biologically Based Treatment

**DOI:** 10.1002/brb3.70757

**Published:** 2025-08-22

**Authors:** Dor Danan, Yaniv Grosskopf, Avi Mayo, Shai Efrati, Ilan Kutz, Erez Lang, Uri Alon, Keren Doenyas‐Barak

**Affiliations:** ^1^ Department of Molecular Cell Biology Weizmann Institute of Science Rehovot Israel; ^2^ Sagol Center for Hyperbaric Medicine and Research Shamir MC Tzrifin Israel; ^3^ Tel Aviv School of Medicine Tel‐Aviv University Tel Aviv‐Yafo Israel

## Abstract

**Objective:**

Emerging evidence suggests that hyperbaric oxygen therapy (HBOT) promotes neuroplasticity and alleviates symptoms in individuals with post‐traumatic stress disorder (PTSD). As a biologically based treatment, HBOT may demonstrate a threshold effect, wherein sufficient treatment leads to sustained symptom improvement, while insufficient treatment results in diminishing benefits. This study tested the threshold hypothesis reanalyzing the end of treatment and a 3‐month follow‐up symptoms scores of a randomized controlled trial (RCT) comparing HBOT to sham treatment in 56 male veterans with treatment‐resistant PTSD.

**Methods:**

A post hoc analysis was conducted on data from the RCT. The elbow method identifies thresholds in the relationship between end‐of‐treatment improvement and follow‐up outcomes. Spearman's rank correlation was used to assess the relationship between changes in cluster‐specific symptoms and total CAPS scores at the end of treatment and at follow‐up evaluation.

**Results:**

Participants achieving ≥ 35% improvement by the end of treatment demonstrated continued improvement at follow‐up (*p* = 2e^−6^). Significant correlations were observed between changes in each of the symptom clusters and total CAPS scores, with the strongest correlation in intrusive symptoms (Cluster B, *r* = 0.74, *p* = 10e^−4^ and *r* = 0.80, *p* = 10e^−4^). Changes in avoidance (Cluster C, *r* = 0.70, *p* = 10e^−4^) at the end of treatment were the best predictors of follow‐up improvements.

**Conclusions:**

A threshold effect is evident in HBOT treatment for PTSD, where ≥ 35% improvement posttreatment predicts continued gains. Symptom reduction beyond the threshold may serve as a target for HBOT when prescribed for PTSD. The suggested underlying physiological mechanisms, involving bistable circuit dynamics, justify evaluating potential threshold effect in other treatment modalities.

**Trial Registration**: ClinicalTrial.gov identifier: NCT04518007

## Graphical abstract

This post hoc analysis of an RCT in veterans with PTSD reveals a threshold effect of HBOT, where ≥ 35% symptom reduction predicts sustained improvement. Cluster‐specific symptom changes—especially avoidance and intrusion—correlated with long‐term outcomes, supporting HBOT's role as a biologically based treatment with neuroplastic potential.

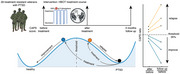

## 1 Introduction

Post‐traumatic stress disorder (PTSD) is a long‐term consequence of exposure to traumatic or stressful events, such as combat, accidents, assault, terror attacks, and natural disasters. PTSD affects up to 30% of combatants (Marmar et al. 2015) and has a major effect on quality of life and on social and occupational function (Schnurr et al. 2009; Pacella et al. 2013). It is characterized by a range of symptoms, which are grouped into four clusters: intrusive symptoms such as nightmares, flashbacks, and recollection of the traumatic event (Cluster B); avoidance behavior (Cluster C); changes in cognition and mood (Cluster D); hypervigilance and hyperarousal (Cluster E) (Edition 2013).

PTSD is often resistant to treatment, with approximately 50% of patients not responding to guideline‐recommended therapies (Friedman 2013; Etkin et al. 2019). Over the past decade, research has highlighted enduring changes in brain activity and structure as key contributors to treatment resistance. Therefore, neuromodulating and biologically based treatment approaches have gained more attention in recent years. One set of approaches such as transcranial magnetic stimulation (TMS) and transcranial electrical (tES) use direct stimulation of specific brain regions to modulate neural activity, with promising effects especially on mood and arousal symptoms (Kan et al. 2020). Dysregulation of the hypothalamic‐pituitary‐adrenal (HPA) axis is also well documented in individuals with PTSD and may represent a potential target for biological intervention (Saccenti et al. 2024), an approach currently in the research phase.

**FIGURE 1:**
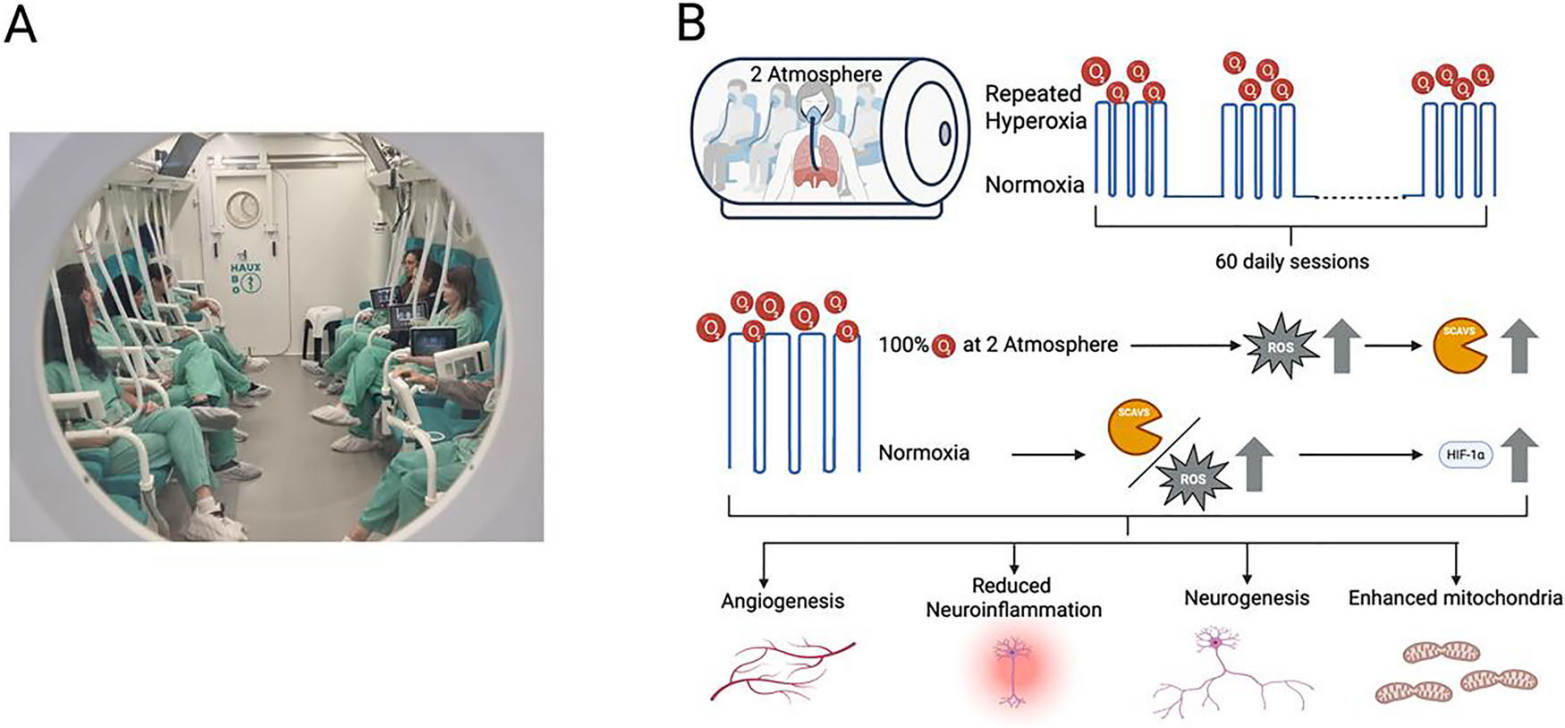
A picture of a 12‐seat multiplace chamber (A), accompanied by a schematic explanation of its biological mechanisms (B). A hyperbaric chamber is used to increase ambient pressure to 2 ATA. The chamber is compressed with air, and once the target pressure is reached, a mask is applied to deliver 100% oxygen. The high oxygen concentration leads to a transient increase in reactive oxygen species (ROS), which in turn induces the production of antioxidant scavenger proteins. During the 90‐min session, the mask is removed for 5 min every 20 min, causing a sharp drop in oxygen levels back to normoxia. At the cellular level, this return to normoxia is perceived as hypoxia due to the imbalance between the rapidly vanishing ROS and the persistently elevated scavenger proteins. This paradoxical, repeated hypoxic signal—induced by cycles of hyperoxia—leads to the upregulation of hypoxia‐inducible factor 1‐alpha (HIF‐1α), which activates genes involved in cellular repair. These mechanisms collectively promote stem cell proliferation, angiogenesis, reduced neuroinflammation, and improved mitochondrial function.

An emerging biological approach to treatment‐resistant PTSD is hyperbaric oxygen therapy (HBOT) (Doenyas‐Barak et al. 2022, 2023a, 2024; Doenyas‐Barak et al. 2023). HBOT uses changes in barometric pressure and gas composition to induce biological effects. In the context of regenerative medicine, including PTSD, HBOT is used to trigger the hyperoxic–hypoxic paradox. The paradox is based on the fact that repeated exposure to elevated oxygen levels followed by a return to normoxia, is perceived at the cellular level as hypoxia, which contributes to the elevation of hypoxia‐inducible factor (HIF) (Hadanny and Efrati 2020). HIF, in turn, activates genes involved in cellular repair. The same mechanisms contributes to stem cell proliferation (Cimino et al. 2012), angiogenesis, reduced neuroinflammation, and improved mitochondrial function (Figure 1).

These repair mechanisms are thought to overcome barriers to tissue recovery, as documented in nonhealing diabetic wounds, in the brain sequelae of stroke and traumatic brain injury, and more recently in PTSD (Doenyas‐Barak et al. 2022, 2023a, 2024; Doenyas‐Barak et al. 2023). A typical treatment course for brain pathology consists of 40–60 daily sessions.

Over the past decade, we have conducted clinical trials demonstrating that veterans with treatment‐resistant PTSD showed positive responses to a course of HBOT (Doenyas‐Barak et al. 2024). Alongside the short‐term effects of HBOT, our previous findings demonstrated a sustained positive impact at 2‐year follow‐up in the majority of the patients, with further improvements in functional outcomes such as occupational status, marital status, and reductions in benzodiazepine and cannabis use. In a recent study, we demonstrated a significant reduction in symptom load in 68% of veterans with treatment‐resistant, combat‐associated PTSD following HBOT compared to sham treatment, with benefits persisting at the 3‐month follow‐up (Doenyas‐Barak et al. 2024). In two of the clinical studies functional MRI was used to demonstrate recovery of the characteristic malfunctioning brain regions.

An effective, disease‐modifying treatment for PTSD should induce a sustained effect because short‐term symptom improvement may be related to participation effects or therapist–patient interactions. As a biologically based treatment that was shown to enhance brain plasticity, a sufficiently effective course of HBOT is expected to promote continued improvement, reflecting the overcoming of barriers to recovery, as demonstrated while treating a nonhealing wound. In contrast, a more modest or insufficient treatment may lead to a decline in improvement over time. This suggests a threshold for treatment‐related improvement: above this threshold, benefits continue to develop, while below it, improvements diminish. We refer to this concept as the threshold hypothesis.

In a recent randomized controlled trial, we have shown significant effect of 60 daily HBOT versus sham sessions on post‐traumatic symptoms in veterans with treatment resistant PTSD (Doenyas‐Barak et al. 2024). The treatment effect was evaluated after the treatment course cassation and at 3‐month follow‐up. Here, we test the threshold hypothesis in HBOT for PTSD by analyzing the clinical scores after treatment and at the 3‐month follow‐up. Furthermore, to better understand the threshold effect, we tested the possibility that the threshold is associated with specific symptom clusters rather than overall improvement. Thus, we tested which of the clusters of PTSD symptoms best associates with overall improvement.

## 2 Methods

### 2.1 HBOT Clinical Trial

The study was a post hoc analysis of a clinical trial described in Doenyas‐Barak et al. (2024). that evaluated the effects of HBOT versus sham treatment on veterans with treatment‐resistant PTSD. Participants were defined as having treatment resistant PTSD if they had residual debilitating PTSD symptoms after being treated with at least one course of trauma‐focused psychotherapy and pharmacotherapy, and still fulfilled the Clinician‐Administered PTSD Scale‐V (CAPS‐V) PTSD diagnostic criteria for PTSD with a score over 20. Briefly, participants were randomly assigned to either an active treatment group or sham. The active treatment group received 60 daily HBOT sessions with 90 min of exposure to 100% oxygen at 2 ATA, with 5‐min air breaks every 20 min. The sham treatment group received 60 daily HBOT sessions with 90 min of exposure to 21% oxygen (room air) at 1.02 ATA, with 5‐min air breaks every 20 min. Participants could not significantly tell whether they were in sham or active groups by questionnaire. Twenty‐eight patients completed the study in each of the treatment arms.

Symptom severity was assessed using the CAPS inventory, conducted at baseline, at the end of the treatment course, and 3 months posttreatment. CAPS‐V was used, a structured interview‐based test that consists of 30 items. Items are rated on a 0–4 severity scale. Note that 20 of the 30 items are summed to give a score that reflects the severity of DSM‐V PTSD symptoms. The total score ranges between 0 and 80, with higher scores indicating more severe PTSD symptoms. The items that contribute to the total CAPS score are organized into four symptom clusters: five items for *intrusion symptoms* (e.g., flashbacks and nightmares), two items for *avoidance symptoms* (e.g., avoiding trauma‐related thoughts or places), seven items for *negative alterations in cognition and mood* (e.g., negative beliefs about oneself or the world and detachment from others), and six items for *arousal and reactivity symptoms* (e.g., hypervigilance and exaggerated startle response).

### 2.2 Data Processing and Analysis

To compare relative improvement, CAPS scores posttreatment and at follow‐up were normalized to CAPS score before treatment for each participant.

To determine a threshold effect, we have used the elbow method. We scanned potential improvement thresholds (between 1 and 0) and evaluated the fraction of participants with post/pre caps scores below the threshold that improved at follow‐up (follow‐up/pre<post/pre). We compared this to 1000 randomized samples where follow‐up/pre scores were shuffled between participants so that they were independent of the post/pre scores. We evaluated the improvement threshold by the threshold at which the distance between the measured and mean randomized curves was maximal.

*Correlation analysis*: Spearman correlation was used to associate each of the cluster scores with overall improvement at the end of the treatment and follow‐up. Significance was evaluated by the bootstrap method and Mann–Whitney tests.

## 3 Results

Baseline patients’ characteristics and CAPS scores are summarized in Table 1.

**TABLE 1:** Baseline characteristics.

	HBOT	SHAM
Age (y)	37.75 ± 8.29	36.4±7.36
Marital status		
Single	9 (32.1)	13 (46.4)
Married	15 (53.6)	15 (53.6)
Divorced	4 (14.3)	0
No. of children	3.05 ± 2	2.6 ± 1.3
Education (y)	13.85 ± 2.89	14 ± 3.4
Working	17 (60.7)	13 (46.4)
Service duration (y)	7.84 ± 8.3	7.2 ± 2.6
Time from last combat exposure	12.4 ± 7.62	12.0 ± 6.8
CAPS at baseline	42.57 ± 9.28	45.11 ± 8.98
Moderate (23–34) (%)	5 (18)	4 (14)
Severe (35–50) (%)	17 (61)	16 (56)
Extreme (51–80) (%)	6 (21)	8 (29)
DASS‐21 at baseline		
Depression	22 ± 8.8	22.7 ± 9.14
Anxiety	20.28 ± 10.38	19.36 ± 10.8
Stress	28.58 ± 9.14	30.64 ± 9.42
Number of sessions completed	59.92 (58–60)	52.42 (20–60)

### 3.1 An Improvement of Over 35% in CAPS Score Shortly After HBOT Is Associated With Further Improvement at 3‐Month Follow‐Up

To test the relation between end of treatment and follow‐up improvement, we asked what fraction of participants which exceeded an improvement of *X*% at the end of treatment showed further improvement at 3‐month follow‐up (Figure 1A). We found that those that improved by more than 35% tended to further improve at follow‐up evaluation (*p* = 2 10e^−6^ using a bootstrap test) (Figure 1B).

In the sham treatment group only one subject showed >35% improvement at posttreatment evaluation compared to baseline, and the improvement attenuated at follow‐up.

### 3.2 Avoidance (Cluster C) Posttreatment Shows the Highest Correlation With Overall Improvement at Follow‐Up

To evaluate which symptom cluster best predicts the follow‐up CAPS score, we evaluated the correlation between the relative change in total CAPS score at follow‐up (follow‐up/post) to the change in each cluster of symptoms at the end of treatment (post/pre). We find that change in avoidance symptoms (Cluster C) at the end of treatment has the highest correlation with CAPS at follow‐up (follow‐up/pre) (*r* = 0.7, *p* = 10e^−4^) (Figure 3c). Intrusive symptoms (Cluster B) and cognitive symptoms (Cluster D) showed milder correlation with follow‐up CAPS improvement (*r* = 0.61, *p* = 0.0005 and *r* = 0.48, *p* = 0.0092, respectively), whereas the hypervigilance and hyperarousal at the end of the treatment (post/pre) showed no significant correlation with the CAPS score at follow‐up (*p* = 0.13) (Figure 3c). Thus, improvement in avoidance (Cluster C) posttreatment is the cluster that best correlated with further improvement of total score at follow‐up.

### 3.3 Despite the Daily Nature of the Intervention, Avoidance (Cluster C) Improved in HBOT and Not in Sham

Improvement in avoidance symptoms (Cluster C) was observed in all but one participant in the HBOT group, with 86% showing further improvement at follow‐up. In contrast, only five out of 28 patients in the sham group showed improvement at the end of treatment, with this improvement diminishing in three of those participants at follow‐up. Thus, improvement in avoidance symptoms is not due to the prosocial aspects of the intervention.

### 3.4 Absolute CAPS Score at Baseline Could Not Predict CAPS Improvement Posttreatment or Improvement at Follow‐Up

To evaluate whether baseline symptom severity could predict treatment outcome, we examined the correlation between baseline CAPS scores and CAPS improvement at the end of treatment and at follow‐up. We found no significant correlation between baseline CAPS score and symptom improvement at either time point (end of treatment: *R* = 0.12, *p* = 0.56; follow‐up: *R* = 0.18, *p* = 0.3).

We then aimed to assess the relationship between baseline severity and symptom trajectory by comparing baseline CAPS scores between two groups: those whose CAPS score at follow‐up was lower (improved) than at the end of treatment, and those whose CAPS score at follow‐up was higher (worsened). We find no significant difference of baseline CAPS scores between the two groups (Figure S1). Thus, symptom severity score at baseline could not predict a favorable trajectory at follow‐up versus posttreatment.

### 3.5 Improvement in Intrusive Symptoms (Cluster B) Is Most Strongly Correlated With Total CAPS Score Both Posttreatment and at Follow‐Up

**FIGURE 2:**
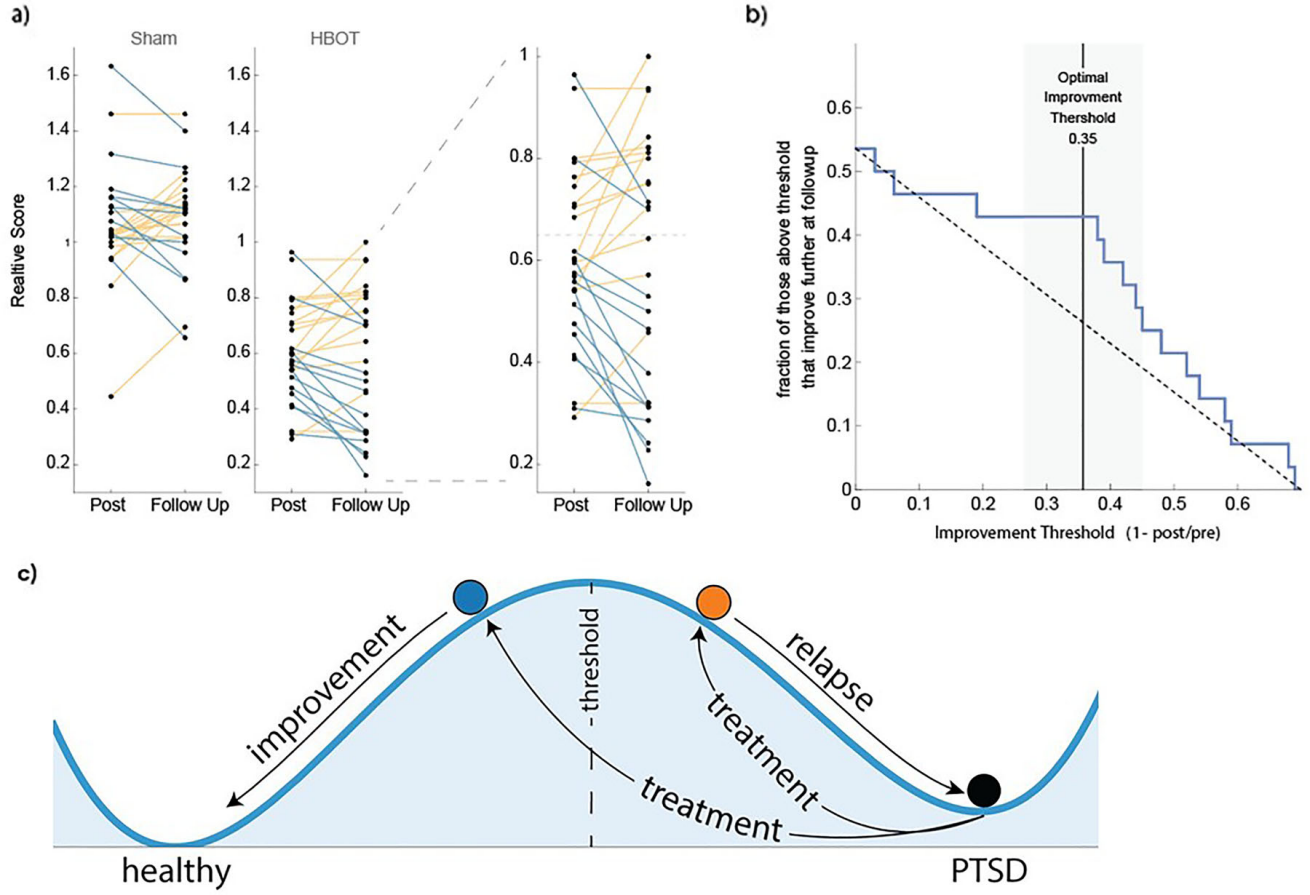
Improvement over 35% after HBOT treatment correlates with further improvement at follow‐up. (a) CAPS scores at the end of treatment and at follow‐up relative to score before treatment for all participants, for sham treatment and HBOT treatment. Blue denotes further improvement at follow‐up, orange denotes no further improvement or worsening at follow‐up. (b) Fraction of HBOT participants with improved CAPS score at follow‐up given posttreatment improvement beyond a threshold. The dashed diagonal line is the fraction expected at random. The optimal improvement threshold, defined as the point of maximal distance between the curve and the diagonal is shown. (c) A threshold effect characterizes biological systems with bistable set points. In the context of PTSD, sufficient treatment may induce a shift in underlying biological processes, leading to symptom reduction and transition to a new set point. However, subthreshold treatment may fail to produce this shift, resulting in a return to the pretreatment state.

**FIGURE 3:**
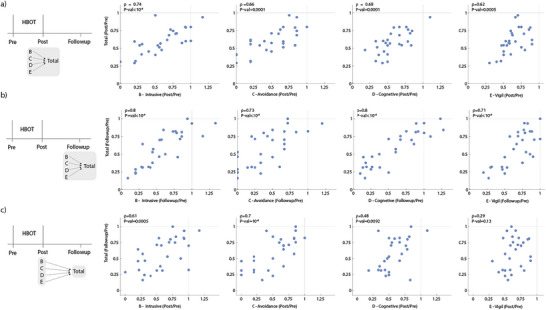
Correlation between symptoms clusters and total CAPS score. Analysis of symptom clusters suggests that avoidance (Cluster C) best predicts follow‐up score, and that intrusive symptoms (Cluster B) are primary components of the total score. (a) Total score versus core of each symptom cluster post treatment. (b) Total score versus score of each symptom cluster at follow‐up. (c) Total score at follow‐up versus each cluster posttreatment. Data are for all HBOT participants, Pearson correlation and p value are shown.

To test which of the symptom clusters best associates with overall improvement after HBOT treatment, we evaluated the correlation between the total CAPS score change (post/pre and follow‐up/pre) to the change in each of the four clusters of symptoms that make up the total score (Figure 3a,b). Because each cluster makes up part of the total score, the correlation is positive. However, different clusters had different correlation strengths. At the end of treatment, intrusive symptoms (Cluster B) showed the strongest correlation to total score(*r* = 0.74,*p* = 10e^−4^). At follow‐up, intrusive symptoms (Class B) and cognitive and mood changes (Cluster D) showed the strongest correlation with the total CAPS score (*r* = 0.8, *p* = 10e^−4^ for both) (Figure 2). We concluded that improvement in intrusive symptoms (Cluster B) best associates with overall CAPS improvement.

## 4 Discussion

We evaluated the relative change in CAPS scores at the end of a 60‐day HBOT or sham treatment course and at a 3‐month follow‐up, identifying a threshold effect. Participants who showed more than a 35% improvement posttreatment tended to continue to improve at follow‐up, whereas those with less than a 35% improvement did not exhibit further gains.

Treatment resistance and poor therapeutical outcomes in PTSD have been linked to specific neurobiological and physiological abnormalities. These include elevated inflammatory markers such as IL‐6; reduced activation and connectivity in fronto‐limbic circuits, particularly between the prefrontal cortex and limbic structures (Friedman 2013; Zilcha‐Mano et al. 2020); and structural and functional impairments in the hippocampus, including reduced volume and altered contextual processing (Duval et al. 2020). These markers have been associated with poor response to trauma‐focused therapies, suggesting they may underlie the mechanisms of treatment resistance.

HBOT has been shown to directly modulate many of these treatment‐resistant biological signatures. It reduces systemic inflammation (Chen et al. 2024), enhances functional connectivity and activation within circuits implicated in PTSD treatment resistance (Doenyas‐Barak et al. 2023a; Doenyas‐Barak et al. 2024), and improves hippocampal blood flow and network integration (Doenyas‐Barak et al. 2024; Catalogna et al. 2022; Ma et al. 2021; Amir et al. 2020). These effects occur in a dose‐dependent manner (Efrati and Ben‐Jacob 2014). The convergence of these systems‐level changes may help explain the threshold‐like clinical improvements observed following a course of HBOT. Thus, HBOT may enable suppressed neurobiological processes to resume, crossing a tipping point beyond which sustained clinical improvement becomes possible.

A second clue to HBOT's mechanism is the possibility of continued biological activity after treatment ends. A similar effect is seen in HBOT for peripheral wounds, where treatment is stopped once new vasculature and granulation tissue are visible, yet healing continues posttreatment until recovery is complete. The threshold effect observed here suggests that similar processes may be engaged in treating the “wounds” of PTSD. These mechanisms could account for the continued improvement observed 3 months after treatment termination at follow‐up.

An alternative explanation for the additional recovery observed 3 months posttreatment is behavioral: A 35% reduction in symptoms at the end of the treatment may be enough to support functioning and encourage positive changes in previous restrictive routines, which in turn, support continued improvement. Exploring threshold effects in other treatment modalities could help clarify the relative contribution of such biological and behavioral mechanisms.

A threshold effect is expected in processes where physiological circuits exhibit bistability (diastasis), as suggested in recent models of major depressive disorder (MDD). A bistable system has two possible steady‐state fixed points—similar to a landscape with two wells separated by a hill (Figure 2c). A strong stimulus such as trauma can push the system from a healthy fixed point over the hill into the pathologic fixed point, in which the system then remains. Treatment can effectively push the system back to its original fixed point, provided the push is strong enough to cross the hill. The threshold represents the minimal strength of the push needed to do so, with weaker effects causing the system to move up the hill (improve posttreatment) but then roll back down toward pathology (worsening at follow‐up).

Interestingly, many clinical trials consistently define a significant treatment effect as a reduction of at least 30% in symptom load, a threshold that might appear arbitrary. However, the findings of the current study provide further support for this threshold, suggesting it may reflect meaningful clinical and biological changes.

We also tested the relation between symptom clusters and HBOT treatment. The CAPS score encompasses four symptom clusters: intrusive symptoms, avoidance, hypervigilance and hyperarousal, and cognitive and mood changes. Among these, the cluster most strongly correlated with improvement posttreatment and at follow‐up was intrusive symptoms (Cluster B). This aligns with the notion that intrusive symptoms are a core feature that is unique to PTSD, stemming from the abnormal acquisition of the traumatic memory (van Marle 2015). Such altered memory acquisition is thought to be fundamental to the mechanism of PTSD. Many PTSD treatment modalities aim to modify or integrate the traumatic memory, thereby alleviating intrusive symptoms (Stickgold 2002). HBOT is known to have a distinct effect on traumatic memory (Doenyas‐Barak et al. 2023b). Consequently, improvements in intrusive symptoms may reflect this process, which can contribute to reductions in overall symptom load.

Improvement of avoidance (Cluster C) at the end of the treatment best predicted further improvement at follow‐up, despite its relative low weight on the overall CAPS score, comprising only two out of 20 items (compared to five, seven, and six items for the other clusters). Reduced avoidance is critical for social and occupational functioning and can enable real‐world “exposure” experiences that are essential for recovery.

One might ask whether HBOT primarily works by reducing avoidance symptoms, given its daily nature over approximately 3 months in an environment that requires interaction with staff and other participants. Notably, while avoidance symptoms significantly improved in the HBOT group, they showed minimal change in the sham group, despite the identical daily routine. This suggests that 3 months of a structured daily routine alone may not be sufficient to address avoidance symptoms in PTSD. We conclude that the benefit of HBOT does not stem from its prosocial daily structure but rather from other effects, which we speculate to involve neuroplastic effects, such as improved mitochondrial function, reduced neuroinflammation, upregulation of brain‐derived neurotrophic factor (BDNF) (Biutifasari et al. 2024), and consequently enhanced activation of regulatory brain regions and improved connectivity, all of which have previously been shown to be induced by HBOT.

The threshold effect observed in this study may provide a meaningful target for therapists utilizing HBOT for PTSD. Achieving a 35% reduction in symptoms by the end of treatment appears to predict a positive trajectory for PTSD symptoms. However, the appropriate strategy for cases where this threshold is not reached requires further study. A recent meta‐analysis indicates a dose‐response relationship for HBOT in PTSD, showing that posttreatment response rates improve with an increased number of sessions and higher barometric pressures. Therefore, prescribing longer treatment courses or higher pressures may be beneficial for patients who do not meet the desired threshold. Alternatively, exploring a different treatment modality might be warranted in such cases.

Limitations of this study include its relatively short follow‐up period of 3 months that may not fully capture the long‐term effects of the treatment. Extending the follow‐up to several years could test the conclusions. Additionally, a sample with greater variability in the causes of PTSD is needed to enhance the generalizability of the findings.

In summary, we find a 35% reduction in symptoms as a threshold for HBOT treatment success, associated with further improvement over time. Reductions in intrusive symptoms best correlate with overall improvement, and improvement in avoidance symptoms best predict continued progress at follow‐up.

## Author Contributions

**Dor Danan**: writing – original draft, investigation. **Yaniv Grosskopf**: investigation, formal analysis, visualization. **Avi Mayo**: investigation. **Shai Efrati**: writing – review and editing. **Ilan Kutz**: writing – review and editing. **Erez Lang**: investigation. **Uri Alon**: conceptualization, writing – review and editing. **Keren Doenyas‐Barak**: conceptualization, writing – review and editing.

## Ethics Statement

The study was conducted in accordance with the Declaration of Helsinki and was approved by Shamir Center's Ethical Review Board.

## Consent

Written informed consent was obtained from all participants prior to inclusion in the study.

## Conflicts of Interest

The authors declare no conflicts of interest.

## Peer Review

The peer review history for this article is available at https://publons.com/publon/10.1002/brb3.70757

## Supporting information




**Supporting Fig.1**:. Absolute CAPS scores do not predict HBOT treatment success. a) Trajectory of total CAPS scores for all participants which worsened at follow‐up. b) Trajectory of total CAPS scores for all participants which improved at follow‐up. c) Initial CAPS scores for the two groups are not drawn from different distributions according to the Mann‐Whitney test. d) Improvement at follow‐up relative to post treatment do not show significant correlation with baseline CAPS scores.

## Data Availability

The data that support the findings of this study are available on request from the corresponding author. The data are not publicly available due to privacy or ethical restrictions.
