# Supplementary figures and images for: Hyperbaric Oxygen Therapy for PTSD: Threshold Effect for Sustained Symptom Improvement in a Biologically Based Treatment
Source: Brain Behav. 2025 Aug 22;15(8):e70757. doi: 10.1002/brb3.70757 (PMC12373514; doi:10.1002/brb3.70757)

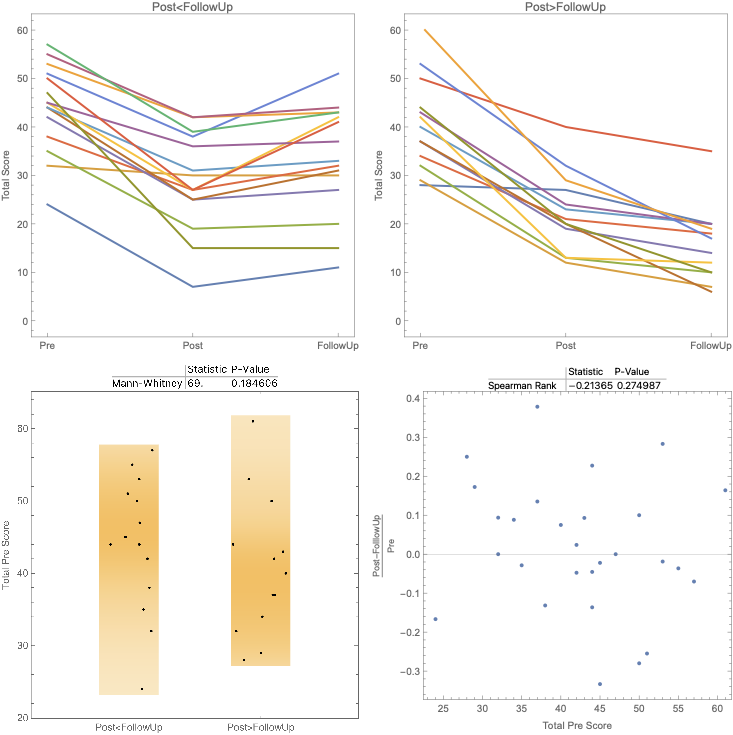


Supplementary Figure 1: Absolute CAPS scores do not predict HBOT treatment success.

Supplement: Supplementary file 1 — Supporting Fig.1:. Absolute CAPS scores do not predict HBOT treatment success. a) Trajectory of total CAPS scores for all participants which worsened at follow‐up. b) Trajectory of total CAPS scores for all participants which improved at follow‐up. c) Initial CAPS scores for the two groups are not drawn from different distributions according to the Mann‐Whitney test. d) Improvement at follow‐up relative to post treatment do not show significant correlation with baseline CAPS scores. [file BRB3-15-e70757-s001.docx]
